# 2-[(*E*)-1,1-Dioxo-2-(2,4,5-tri­fluoro­benz­yl)-3,4-di­hydro-2*H*-1,2-benzo­thia­zin-4-yl­idene]acetic acid

**DOI:** 10.1107/S1600536814013245

**Published:** 2014-06-14

**Authors:** Shaojuan Zhu, Shagufta Parveen, Changjin Zhu

**Affiliations:** a School of Chemical Engineering and Environment, Beijing Institute of Technology, Beijing 100081, People’s Republic of China

## Abstract

In the asymmetric unit of the title compound, C_17_H_12_F_3_NO_4_S, there are two conformationally similar mol­ecules in which the heterocyclic thia­zine ring adopts a half-chair conformation, with the dihedral angle between the two benzene rings being 24.84 (8) and 37.42 (8)°. In the crystal, the mol­ecules form dimers through cyclic carb­oxy­lic acid O—H⋯O hydrogen-bonding associations [graph set *R* ^2^_2_(8)] and are extended into chains along [101] through weak C—H⋯O_sulfin­yl_ hydrogen bonds [graph set *R* ^2^_2_(14)]..

## Related literature   

For pharmaceutical and biological properties of 1,2-benzo­thia­zines, see: Zia-ur-Rehman *et al.* (2005 ▶); Lombardino *et al.* (1971 ▶); Bihovsky *et al.* (2004 ▶); For synthetic details of the title compound, see: Parveen *et al.* (2014*b* ▶). For related structures, see: Yang *et al.* (2012 ▶); Parveen *et al.* (2014*a* ▶). For graph-set analysis, see: Etter *et al.* 1990 ▶).
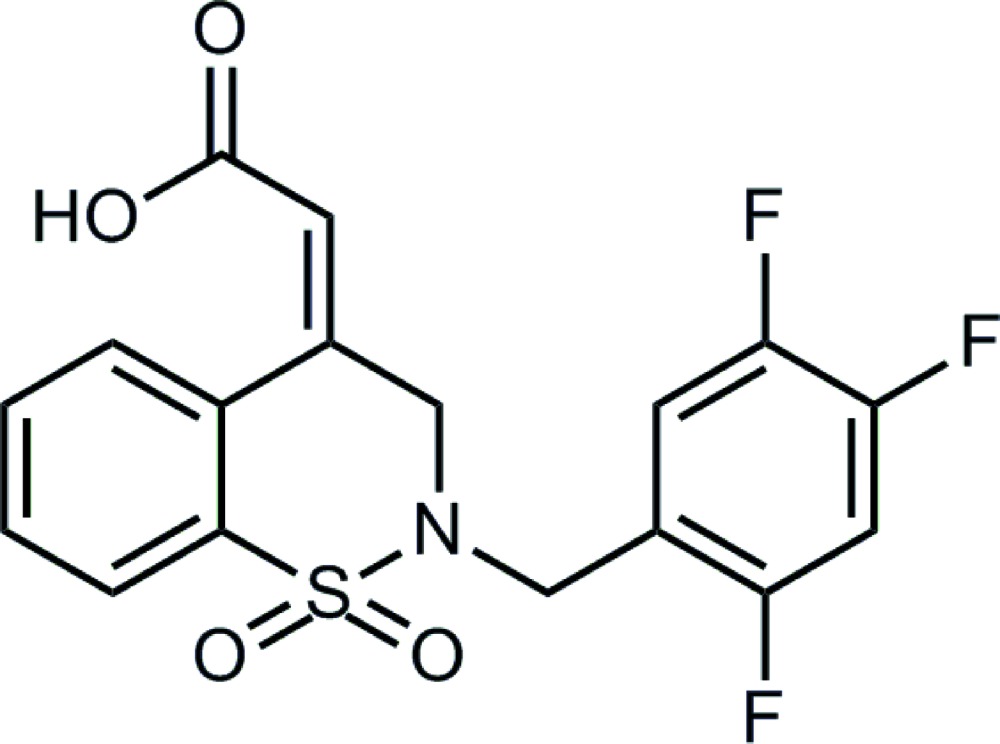


## Experimental   

### 

#### Crystal data  

C_17_H_12_F_3_NO_4_S
*M* *_r_* = 383.34
Triclinic, 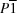

*a* = 8.0028 (10) Å
*b* = 14.249 (2) Å
*c* = 15.076 (2) Å
α = 104.631 (8)°
β = 99.915 (6)°
γ = 104.237 (6)°
*V* = 1561.2 (4) Å^3^
*Z* = 4
Mo *K*α radiation
μ = 0.27 mm^−1^
*T* = 153 K
0.31 × 0.26 × 0.18 mm

#### Data collection  

Rigaku AFC10/Saturn724+ CCD-detector diffractometer
Absorption correction: multi-scan (*CrystalClear*; Rigaku, 2008 ▶) *T*_min_ = 0.932, *T*_max_ = 0.964
20894 measured reflections
8293 independent reflections
6954 reflections with *I* > 2σ(*I*)
*R*_int_ = 0.030

#### Refinement  

*R*[*F* ^2^ > 2σ(*F* ^2^)] = 0.042
*wR*(*F* ^2^) = 0.114
*S* = 1.00
8293 reflections
477 parameters
H atoms treated by a mixture of independent and constrained refinement
Δρ_max_ = 0.32 e Å^−3^
Δρ_min_ = −0.43 e Å^−3^

### 

Data collection: *CrystalClear* (Rigaku, 2008 ▶); cell refinement: *CrystalClear* (Rigaku, 2008 ▶); data reduction: *CrystalClear*; program(s) used to solve structure: *SHELXS97* (Sheldrick, 2008 ▶); program(s) used to refine structure: *SHELXL97* (Sheldrick, 2008 ▶); molecular graphics: *DIAMOND* (Brandenburg, 1998 ▶); software used to prepare material for publication: *CrystalStructure* (Rigaku, 2008 ▶).

## Supplementary Material

Crystal structure: contains datablock(s) I, New_Global_Publ_Block. DOI: 10.1107/S1600536814013245/zs2301sup1.cif

Structure factors: contains datablock(s) I. DOI: 10.1107/S1600536814013245/zs2301Isup2.hkl

Supporting information file. DOI: 10.1107/S1600536814013245/zs2301Isup3.mol

Supporting information file. DOI: 10.1107/S1600536814013245/zs2301Isup4.cml

CCDC reference: 1007103

Additional supporting information:  crystallographic information; 3D view; checkCIF report

## Figures and Tables

**Table 1:** Hydrogen-bond geometry (Å, °)

*D*—H⋯*A*	*D*—H	H⋯*A*	*D*⋯*A*	*D*—H⋯*A*
O3—H3*O*⋯O8^i^	0.85 (3)	1.86 (3)	2.7063 (17)	172 (3)
O7—H7*O*⋯O4^ii^	0.92 (3)	1.73 (3)	2.6473 (17)	177 (2)
C15—H15⋯O6	0.95	2.34	3.267 (2)	165
C32—H32⋯O2	0.95	2.61	3.539 (2)	165

Symmetry codes: (i) 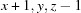; (ii) 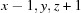.

**Fig. 1.**
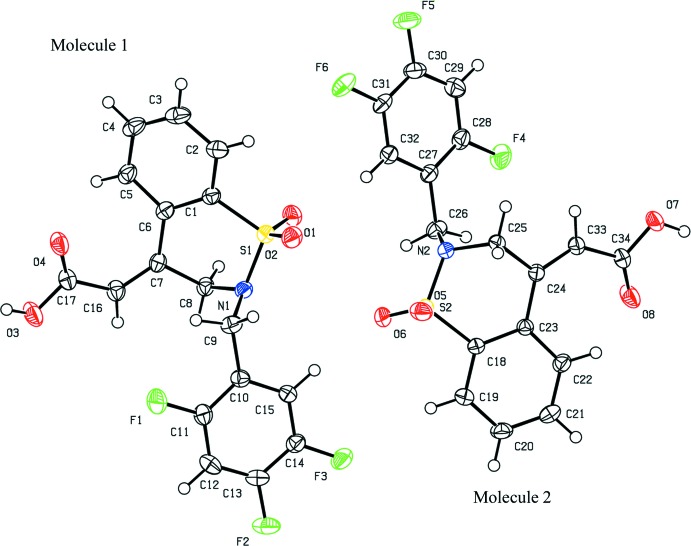
The molecular conformation and atom numbering scheme for the two independent molecules (A and B) of the title compound in the asymmetric unit. Displacement ellipsoids are drawn at the 50% probability level.

**Fig. 2.**
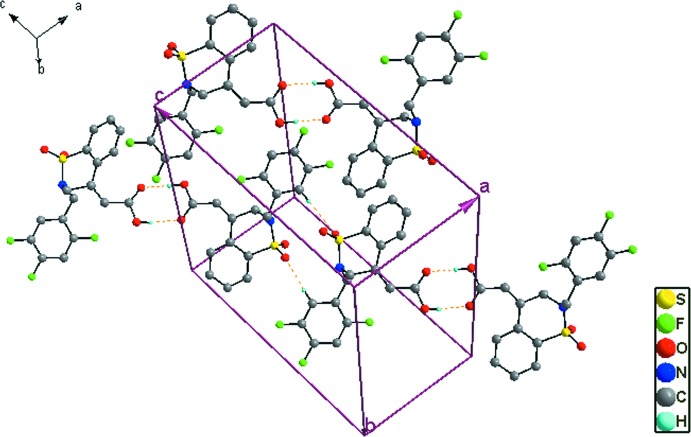
Hydrogen-bonding interactions (O—H···O and C—H···O), shown as dashed lines, in the one-dimensional chain structure of the title compound. H atoms non–participating in hydrogen-bonding are omitted for clarity.

## supplementary crystallographic information

### S1. Comment

1,2-Benzothiazine-1,1-dioxide derivatives are reported as having anti- inflammatory and anti-bacterial activities (Lombardino *et al.*, 1971) while some of its derivatives have been found to be calpain 1 inhibitors (Bihovsky *et al.*, 2004). More recently, its derivatives were reported as aldose reductase inhibitors (Parveen *et al.*, 2014*b*). Herein, we report the structure of the title derivative, C_17_H_12_F_3_NO_4_S, which is the *E* isomer of the previously published isomer 2-[(*Z*)-1,1-dioxo-2-(2,4,5-trifluorobenzyl)-3,4- dihydro-2*H*-1,2-benzothiazin-4-ylidene]acetic acid (Parveen *et al.*, 2014*a*).

In the title compound, there are two conformationally similar molecules (*A* and *B*) in the asymmetric unit (Fig. 1). The dihedral angles between mean plane of the two benzene rings [(C1—C6) and (C10—C15) in *A* and (C18–C23) and (C27–C32) in *B*] are 37.42 (8) and 24.84 (7)°, respectively. These values compare with 43.28 (9)° in the *Z* isomer (Parveen *et al.*, 2014*a*). The heterocyclic thiazine ring adopts a half-chair conformation. The acetic acid substituent groups show only minor conformational differences: torsion angles C8—C7—C16—C17 and C7—C16—C17—O3 [169.7 (16) and 174.59 (17)°, respectively] in *A* campare with C25—C24— C33—C34 and C24—C33—C34—O7 [-179.25 (16) and -168.18 (17), respectively] in *B*.

In the crystal the molecules form centrosymmetric dimers through intermolecular cyclic carboxylic acid O—H···O hydrogen-bonding associations [graph set *R*^2^_2_(8) (Etter *et al.*, 1990)]. These dimers form one-dimensional chains which extend along [101] (Fig. 2), through weak duplex C—H···O_sulfinyl_ hydrogen-bonding associations (Table 1) [graph set *R*^2^_2_(14)].

### S2. Experimental

A mixture of *E*-2-[2-(2,4,5-Trifluorobenzyl)-1,1-dioxido-2*H*-1,2-benzothiazin- 4(3*H*)-ylidene]acetic acid methyl ester (0.5 mmol), 10 *M* hydrochloric acid (8 mL), and 1,4-dioxane (5 mL) was refluxed at 60°C for 2 h. The crude product obtained was washed with cold water (3 times, 10 mL) and purified by flash chromatography with CH_2_Cl_2_ and methanol (100:1) as eluent, which afforded a white solid product on concentration under vacuum. Recrystallization from ethanol gave crystals of the title compound suitable for the X-ray analysis (yield = 60%).

### S3. Refinement

H atoms bonded to O1 and O3 were located from a difference-Fourier map and were refined freely. The remaining H atoms were positioned geometrically, with C—H = 0.95 and 0.99 Å for aromatic and methylene H, respectively, and constrained to ride on their parent atoms with *U*_iso_(H) = 1.2*U*_eq_(C).

### Crystal data

**Table:**

C_17_H_12_F_3_NO_4_S	*Z* = 4
*M**_r_* = 383.34	*F*(000) = 784
Triclinic, *P*1	*D*_x_ = 1.631 Mg m^−^^3^
*a* = 8.0028 (10) Å	Mo *K*α radiation, λ = 0.71073 Å
*b* = 14.249 (2) Å	Cell parameters from 5221 reflections
*c* = 15.076 (2) Å	θ = 2.4–29.1°
α = 104.631 (8)°	µ = 0.27 mm^−^^1^
β = 99.915 (6)°	*T* = 153 K
γ = 104.237 (6)°	Prism, colourless
*V* = 1561.2 (4) Å^3^	0.31 × 0.26 × 0.18 mm

### Data collection

**Table:**

Rigaku AFC10/Saturn724+ CCD-detector diffractometer	8293 independent reflections
Radiation source: Rotating Anode	6954 reflections with *I* > 2σ(*I*)
Graphite monochromator	*R*_int_ = 0.030
Detector resolution: 28.5714 pixels mm^-1^	θ_max_ = 29.1°, θ_min_ = 2.7°
φ and ω scans	*h* = −10→10
Absorption correction: multi-scan (*CrystalClear*; Rigaku, 2008)	*k* = −19→19
*T*_min_ = 0.932, *T*_max_ = 0.964	*l* = −20→20
20894 measured reflections	

### Refinement

**Table:**

Refinement on *F*^2^	Primary atom site location: structure-invariant direct methods
Least-squares matrix: full	Secondary atom site location: difference Fourier map
*R*[*F*^2^ > 2σ(*F*^2^)] = 0.042	Hydrogen site location: inferred from neighbouring sites
*wR*(*F*^2^) = 0.114	H atoms treated by a mixture of independent and constrained refinement
*S* = 1.00	*w* = 1/[σ^2^(*F*_o_^2^) + (0.061*P*)^2^ + 0.226*P*] where *P* = (*F*_o_^2^ + 2*F*_c_^2^)/3
8293 reflections	(Δ/σ)_max_ = 0.001
477 parameters	Δρ_max_ = 0.32 e Å^−^^3^
0 restraints	Δρ_min_ = −0.43 e Å^−^^3^

### Special details

**Table:**

Geometry. All e.s.d.'s (except the e.s.d. in the dihedral angle between two l.s. planes) are estimated using the full covariance matrix. The cell e.s.d.'s are taken into account individually in the estimation of e.s.d.'s in distances, angles and torsion angles; correlations between e.s.d.'s in cell parameters are only used when they are defined by crystal symmetry. An approximate (isotropic) treatment of cell e.s.d.'s is used for estimating e.s.d.'s involving l.s. planes.
Refinement. Refinement of *F*^2^ against ALL reflections. The weighted *R*-factor *wR* and goodness of fit *S* are based on *F*^2^, conventional *R*-factors *R* are based on *F*, with *F* set to zero for negative *F*^2^. The threshold expression of *F*^2^ > σ(*F*^2^) is used only for calculating *R*-factors(gt) *etc*. and is not relevant to the choice of reflections for refinement. *R*-factors based on *F*^2^ are statistically about twice as large as those based on *F*, and *R*-factors based on ALL data will be even larger.

### Fractional atomic coordinates and isotropic or equivalent isotropic displacement parameters (Å^2^)

**Table:**

	*x*	*y*	*z*	*U*_iso_*/*U*_eq_	
S1	0.73455 (5)	0.69761 (3)	0.55702 (3)	0.02391 (10)	
S2	0.50292 (5)	0.87262 (3)	0.79867 (3)	0.02531 (10)	
F1	0.58349 (14)	0.89772 (9)	0.31143 (8)	0.0450 (3)	
F2	0.03716 (14)	0.93851 (8)	0.35868 (8)	0.0423 (3)	
F3	0.08711 (14)	0.88184 (8)	0.51527 (8)	0.0391 (3)	
F4	0.45408 (14)	0.54711 (9)	0.91587 (8)	0.0431 (3)	
F5	1.01396 (15)	0.50243 (9)	0.90295 (8)	0.0469 (3)	
F6	1.06588 (13)	0.63243 (8)	0.80223 (8)	0.0387 (3)	
O1	0.63170 (15)	0.60907 (9)	0.57395 (8)	0.0311 (3)	
O2	0.82039 (16)	0.78775 (9)	0.63550 (8)	0.0328 (3)	
O4	0.99668 (15)	0.69914 (10)	0.24217 (8)	0.0337 (3)	
O3	0.78052 (17)	0.70022 (12)	0.12780 (9)	0.0417 (3)	
O5	0.63875 (15)	0.96931 (9)	0.83329 (9)	0.0335 (3)	
O6	0.44968 (17)	0.82015 (11)	0.69925 (8)	0.0386 (3)	
O7	0.26182 (16)	0.72184 (10)	1.15812 (9)	0.0334 (3)	
O8	0.06107 (15)	0.74619 (10)	1.05181 (9)	0.0379 (3)	
N1	0.60349 (16)	0.72401 (9)	0.47719 (9)	0.0224 (3)	
N2	0.56830 (16)	0.79812 (10)	0.85392 (9)	0.0232 (3)	
C1	0.89250 (19)	0.65910 (11)	0.49927 (11)	0.0233 (3)	
C2	1.0447 (2)	0.65398 (12)	0.55604 (13)	0.0298 (3)	
H2	1.0695	0.6801	0.6230	0.036*	
C3	1.1599 (2)	0.61036 (13)	0.51375 (14)	0.0344 (4)	
H3	1.2670	0.6092	0.5515	0.041*	
C4	1.1179 (2)	0.56859 (13)	0.41639 (14)	0.0341 (4)	
H4	1.1912	0.5334	0.3879	0.041*	
C5	0.9703 (2)	0.57747 (12)	0.35992 (13)	0.0286 (3)	
H5	0.9452	0.5495	0.2931	0.034*	
C6	0.85747 (19)	0.62700 (11)	0.39983 (11)	0.0234 (3)	
C7	0.70501 (19)	0.64394 (11)	0.34115 (11)	0.0232 (3)	
C8	0.54758 (19)	0.64252 (11)	0.38476 (11)	0.0238 (3)	
H8A	0.4509	0.6533	0.3417	0.029*	
H8B	0.5017	0.5755	0.3940	0.029*	
C9	0.6528 (2)	0.82967 (12)	0.47205 (12)	0.0273 (3)	
H9A	0.7281	0.8352	0.4268	0.033*	
H9B	0.7224	0.8774	0.5350	0.033*	
C10	0.4876 (2)	0.85800 (11)	0.44047 (11)	0.0237 (3)	
C11	0.4596 (2)	0.89144 (12)	0.36246 (12)	0.0291 (3)	
C12	0.3112 (2)	0.91996 (13)	0.33337 (12)	0.0320 (4)	
H12	0.2965	0.9430	0.2795	0.038*	
C13	0.1865 (2)	0.91365 (12)	0.38516 (12)	0.0285 (3)	
C14	0.2105 (2)	0.88278 (12)	0.46446 (12)	0.0266 (3)	
C15	0.3584 (2)	0.85423 (11)	0.49217 (11)	0.0245 (3)	
H15	0.3727	0.8319	0.5465	0.029*	
C16	0.6974 (2)	0.66706 (12)	0.25983 (11)	0.0274 (3)	
H16	0.5849	0.6697	0.2297	0.033*	
C17	0.8406 (2)	0.68902 (12)	0.21090 (11)	0.0273 (3)	
C18	0.31608 (19)	0.89025 (11)	0.83939 (11)	0.0224 (3)	
C19	0.2095 (2)	0.93054 (13)	0.78660 (12)	0.0291 (3)	
H19	0.2374	0.9444	0.7315	0.035*	
C20	0.0627 (2)	0.95032 (13)	0.81459 (12)	0.0304 (3)	
H20	−0.0118	0.9772	0.7787	0.036*	
C21	0.0259 (2)	0.93060 (12)	0.89503 (12)	0.0282 (3)	
H21	−0.0744	0.9447	0.9147	0.034*	
C22	0.1323 (2)	0.89045 (11)	0.94806 (11)	0.0257 (3)	
H22	0.1043	0.8787	1.0038	0.031*	
C23	0.27989 (18)	0.86690 (10)	0.92127 (10)	0.0199 (3)	
C24	0.39674 (19)	0.82390 (11)	0.97612 (10)	0.0207 (3)	
C25	0.57919 (19)	0.83583 (12)	0.95578 (10)	0.0225 (3)	
H25A	0.6501	0.9087	0.9807	0.027*	
H25B	0.6418	0.7981	0.9894	0.027*	
C26	0.4900 (2)	0.68674 (12)	0.80941 (12)	0.0276 (3)	
H26A	0.4519	0.6705	0.7398	0.033*	
H26B	0.3843	0.6616	0.8324	0.033*	
C27	0.6278 (2)	0.63582 (11)	0.83449 (11)	0.0237 (3)	
C28	0.6062 (2)	0.56868 (12)	0.88642 (12)	0.0286 (3)	
C29	0.7309 (2)	0.52137 (13)	0.91045 (13)	0.0346 (4)	
H29	0.7113	0.4752	0.9461	0.042*	
C30	0.8843 (2)	0.54411 (13)	0.88054 (12)	0.0316 (4)	
C31	0.9114 (2)	0.61120 (12)	0.82925 (11)	0.0266 (3)	
C32	0.7862 (2)	0.65739 (11)	0.80590 (11)	0.0249 (3)	
H32	0.8071	0.7037	0.7705	0.030*	
C33	0.3679 (2)	0.77728 (11)	1.04221 (11)	0.0242 (3)	
H33	0.4672	0.7587	1.0681	0.029*	
C34	0.2146 (2)	0.74921 (11)	1.08260 (11)	0.0250 (3)	
H7O	0.168 (3)	0.7121 (19)	1.1855 (17)	0.067 (8)*	
H3O	0.865 (4)	0.720 (2)	0.1031 (19)	0.077 (9)*	

### Atomic displacement parameters (Å^2^)

**Table:**

	*U*^11^	*U*^22^	*U*^33^	*U*^12^	*U*^13^	*U*^23^
S1	0.02321 (19)	0.02732 (19)	0.0232 (2)	0.00954 (14)	0.00707 (15)	0.00851 (14)
S2	0.02388 (19)	0.0386 (2)	0.0241 (2)	0.01631 (16)	0.01081 (15)	0.01787 (16)
F1	0.0420 (6)	0.0616 (7)	0.0486 (7)	0.0182 (5)	0.0274 (5)	0.0332 (6)
F2	0.0334 (6)	0.0458 (6)	0.0555 (7)	0.0206 (5)	0.0046 (5)	0.0252 (5)
F3	0.0347 (5)	0.0464 (6)	0.0510 (7)	0.0214 (5)	0.0237 (5)	0.0229 (5)
F4	0.0389 (6)	0.0487 (6)	0.0551 (7)	0.0143 (5)	0.0289 (5)	0.0267 (5)
F5	0.0471 (7)	0.0532 (7)	0.0505 (7)	0.0352 (6)	0.0067 (5)	0.0174 (6)
F6	0.0234 (5)	0.0409 (6)	0.0516 (7)	0.0125 (4)	0.0154 (5)	0.0070 (5)
O1	0.0305 (6)	0.0368 (6)	0.0326 (6)	0.0106 (5)	0.0128 (5)	0.0184 (5)
O2	0.0334 (6)	0.0353 (6)	0.0253 (6)	0.0123 (5)	0.0042 (5)	0.0023 (5)
O4	0.0260 (6)	0.0490 (7)	0.0280 (6)	0.0065 (5)	0.0093 (5)	0.0182 (5)
O3	0.0297 (7)	0.0699 (10)	0.0330 (7)	0.0114 (6)	0.0110 (5)	0.0304 (7)
O5	0.0253 (6)	0.0410 (7)	0.0467 (8)	0.0122 (5)	0.0143 (5)	0.0293 (6)
O6	0.0427 (7)	0.0659 (9)	0.0222 (6)	0.0336 (7)	0.0137 (5)	0.0188 (6)
O7	0.0327 (6)	0.0492 (7)	0.0321 (7)	0.0171 (6)	0.0153 (5)	0.0269 (6)
O8	0.0249 (6)	0.0608 (8)	0.0334 (7)	0.0077 (6)	0.0080 (5)	0.0283 (6)
N1	0.0209 (6)	0.0236 (6)	0.0241 (6)	0.0081 (5)	0.0066 (5)	0.0075 (5)
N2	0.0228 (6)	0.0309 (7)	0.0217 (6)	0.0132 (5)	0.0079 (5)	0.0112 (5)
C1	0.0214 (7)	0.0221 (7)	0.0293 (8)	0.0075 (5)	0.0089 (6)	0.0104 (6)
C2	0.0246 (8)	0.0301 (8)	0.0381 (9)	0.0092 (6)	0.0062 (7)	0.0166 (7)
C3	0.0222 (8)	0.0323 (8)	0.0563 (12)	0.0104 (7)	0.0107 (8)	0.0239 (8)
C4	0.0278 (8)	0.0303 (8)	0.0588 (12)	0.0144 (7)	0.0246 (8)	0.0240 (8)
C5	0.0290 (8)	0.0259 (7)	0.0379 (9)	0.0102 (6)	0.0182 (7)	0.0136 (7)
C6	0.0215 (7)	0.0198 (6)	0.0310 (8)	0.0056 (5)	0.0105 (6)	0.0094 (6)
C7	0.0211 (7)	0.0229 (7)	0.0237 (7)	0.0047 (5)	0.0073 (6)	0.0050 (6)
C8	0.0208 (7)	0.0268 (7)	0.0237 (8)	0.0063 (6)	0.0070 (6)	0.0076 (6)
C9	0.0226 (7)	0.0239 (7)	0.0368 (9)	0.0076 (6)	0.0074 (6)	0.0109 (6)
C10	0.0236 (7)	0.0205 (7)	0.0272 (8)	0.0060 (6)	0.0058 (6)	0.0088 (6)
C11	0.0287 (8)	0.0303 (8)	0.0325 (9)	0.0071 (6)	0.0133 (7)	0.0148 (7)
C12	0.0360 (9)	0.0315 (8)	0.0309 (9)	0.0082 (7)	0.0050 (7)	0.0180 (7)
C13	0.0249 (8)	0.0239 (7)	0.0367 (9)	0.0096 (6)	0.0013 (7)	0.0115 (6)
C14	0.0253 (7)	0.0252 (7)	0.0318 (9)	0.0089 (6)	0.0104 (6)	0.0100 (6)
C15	0.0273 (8)	0.0247 (7)	0.0255 (8)	0.0100 (6)	0.0079 (6)	0.0115 (6)
C16	0.0232 (7)	0.0315 (8)	0.0257 (8)	0.0049 (6)	0.0068 (6)	0.0085 (6)
C17	0.0278 (8)	0.0293 (8)	0.0235 (8)	0.0048 (6)	0.0073 (6)	0.0091 (6)
C18	0.0208 (7)	0.0274 (7)	0.0230 (7)	0.0107 (6)	0.0068 (6)	0.0105 (6)
C19	0.0292 (8)	0.0383 (9)	0.0268 (8)	0.0171 (7)	0.0073 (6)	0.0153 (7)
C20	0.0257 (8)	0.0353 (8)	0.0329 (9)	0.0160 (7)	0.0028 (7)	0.0117 (7)
C21	0.0200 (7)	0.0288 (8)	0.0360 (9)	0.0102 (6)	0.0077 (6)	0.0073 (7)
C22	0.0247 (7)	0.0261 (7)	0.0307 (8)	0.0099 (6)	0.0119 (6)	0.0107 (6)
C23	0.0191 (7)	0.0207 (6)	0.0205 (7)	0.0062 (5)	0.0048 (5)	0.0071 (5)
C24	0.0205 (7)	0.0223 (7)	0.0197 (7)	0.0065 (5)	0.0060 (5)	0.0063 (5)
C25	0.0209 (7)	0.0293 (7)	0.0214 (7)	0.0105 (6)	0.0059 (6)	0.0117 (6)
C26	0.0208 (7)	0.0298 (8)	0.0302 (8)	0.0085 (6)	0.0042 (6)	0.0065 (6)
C27	0.0213 (7)	0.0260 (7)	0.0234 (8)	0.0086 (6)	0.0063 (6)	0.0050 (6)
C28	0.0273 (8)	0.0301 (8)	0.0313 (9)	0.0090 (6)	0.0135 (7)	0.0101 (6)
C29	0.0439 (10)	0.0333 (9)	0.0340 (9)	0.0171 (8)	0.0124 (8)	0.0162 (7)
C30	0.0331 (9)	0.0328 (8)	0.0307 (9)	0.0194 (7)	0.0021 (7)	0.0075 (7)
C31	0.0196 (7)	0.0283 (8)	0.0293 (8)	0.0093 (6)	0.0075 (6)	0.0019 (6)
C32	0.0244 (7)	0.0254 (7)	0.0260 (8)	0.0084 (6)	0.0086 (6)	0.0077 (6)
C33	0.0240 (7)	0.0288 (7)	0.0228 (7)	0.0089 (6)	0.0080 (6)	0.0106 (6)
C34	0.0282 (8)	0.0263 (7)	0.0219 (7)	0.0067 (6)	0.0080 (6)	0.0102 (6)

### Geometric parameters (Å, º)

**Table:**

S1—O2	1.4282 (12)	C9—H9A	0.9900
S1—O1	1.4347 (12)	C9—H9B	0.9900
S1—N1	1.6365 (13)	C10—C11	1.380 (2)
S1—C1	1.7708 (16)	C10—C15	1.396 (2)
S2—O6	1.4298 (13)	C11—C12	1.384 (2)
S2—O5	1.4342 (13)	C12—C13	1.369 (2)
S2—N2	1.6322 (13)	C12—H12	0.9500
S2—C18	1.7603 (15)	C13—C14	1.374 (2)
F1—C11	1.3553 (18)	C14—C15	1.376 (2)
F2—C13	1.3525 (18)	C15—H15	0.9500
F3—C14	1.3494 (18)	C16—C17	1.478 (2)
F4—C28	1.3578 (18)	C16—H16	0.9500
F5—C30	1.3519 (19)	C18—C19	1.388 (2)
F6—C31	1.3554 (17)	C18—C23	1.415 (2)
O4—C17	1.2156 (19)	C19—C20	1.383 (2)
O3—C17	1.326 (2)	C19—H19	0.9500
O3—H3O	0.85 (3)	C20—C21	1.375 (2)
O7—C34	1.3189 (19)	C20—H20	0.9500
O7—H7O	0.92 (3)	C21—C22	1.389 (2)
O8—C34	1.2224 (19)	C21—H21	0.9500
N1—C8	1.4841 (19)	C22—C23	1.401 (2)
N1—C9	1.4853 (19)	C22—H22	0.9500
N2—C25	1.4725 (19)	C23—C24	1.485 (2)
N2—C26	1.480 (2)	C24—C33	1.351 (2)
C1—C2	1.392 (2)	C24—C25	1.521 (2)
C1—C6	1.407 (2)	C25—H25A	0.9900
C2—C3	1.388 (2)	C25—H25B	0.9900
C2—H2	0.9500	C26—C27	1.508 (2)
C3—C4	1.383 (3)	C26—H26A	0.9900
C3—H3	0.9500	C26—H26B	0.9900
C4—C5	1.385 (2)	C27—C28	1.379 (2)
C4—H4	0.9500	C27—C32	1.397 (2)
C5—C6	1.401 (2)	C28—C29	1.383 (2)
C5—H5	0.9500	C29—C30	1.373 (2)
C6—C7	1.487 (2)	C29—H29	0.9500
C7—C16	1.343 (2)	C30—C31	1.374 (2)
C7—C8	1.516 (2)	C31—C32	1.372 (2)
C8—H8A	0.9900	C32—H32	0.9500
C8—H8B	0.9900	C33—C34	1.475 (2)
C9—C10	1.510 (2)	C33—H33	0.9500
			
O2—S1—O1	118.55 (7)	C13—C14—C15	120.66 (15)
O2—S1—N1	108.96 (7)	C14—C15—C10	120.07 (14)
O1—S1—N1	107.34 (7)	C14—C15—H15	120.0
O2—S1—C1	110.39 (7)	C10—C15—H15	120.0
O1—S1—C1	106.24 (7)	C7—C16—C17	128.74 (15)
N1—S1—C1	104.43 (7)	C7—C16—H16	115.6
O6—S2—O5	118.94 (8)	C17—C16—H16	115.6
O6—S2—N2	108.52 (7)	O4—C17—O3	122.46 (15)
O5—S2—N2	107.54 (7)	O4—C17—C16	125.63 (15)
O6—S2—C18	109.72 (7)	O3—C17—C16	111.87 (14)
O5—S2—C18	107.20 (7)	C19—C18—C23	122.65 (14)
N2—S2—C18	103.89 (7)	C19—C18—S2	115.21 (12)
C17—O3—H3O	111.3 (19)	C23—C18—S2	122.13 (11)
C34—O7—H7O	108.4 (16)	C20—C19—C18	119.63 (15)
C8—N1—C9	115.31 (12)	C20—C19—H19	120.2
C8—N1—S1	111.40 (10)	C18—C19—H19	120.2
C9—N1—S1	117.72 (10)	C21—C20—C19	119.25 (15)
C25—N2—C26	115.52 (12)	C21—C20—H20	120.4
C25—N2—S2	110.86 (10)	C19—C20—H20	120.4
C26—N2—S2	118.25 (10)	C20—C21—C22	121.26 (15)
C2—C1—C6	121.78 (14)	C20—C21—H21	119.4
C2—C1—S1	117.04 (12)	C22—C21—H21	119.4
C6—C1—S1	120.96 (11)	C21—C22—C23	121.56 (15)
C3—C2—C1	119.30 (17)	C21—C22—H22	119.2
C3—C2—H2	120.3	C23—C22—H22	119.2
C1—C2—H2	120.3	C22—C23—C18	115.61 (14)
C4—C3—C2	119.67 (16)	C22—C23—C24	123.11 (13)
C4—C3—H3	120.2	C18—C23—C24	121.24 (13)
C2—C3—H3	120.2	C33—C24—C23	130.34 (14)
C3—C4—C5	120.76 (16)	C33—C24—C25	114.86 (13)
C3—C4—H4	119.6	C23—C24—C25	114.79 (12)
C5—C4—H4	119.6	N2—C25—C24	112.43 (12)
C4—C5—C6	121.02 (16)	N2—C25—H25A	109.1
C4—C5—H5	119.5	C24—C25—H25A	109.1
C6—C5—H5	119.5	N2—C25—H25B	109.1
C5—C6—C1	116.90 (15)	C24—C25—H25B	109.1
C5—C6—C7	122.38 (15)	H25A—C25—H25B	107.8
C1—C6—C7	120.71 (13)	N2—C26—C27	108.89 (12)
C16—C7—C6	127.74 (14)	N2—C26—H26A	109.9
C16—C7—C8	117.96 (14)	C27—C26—H26A	109.9
C6—C7—C8	114.11 (13)	N2—C26—H26B	109.9
N1—C8—C7	109.66 (12)	C27—C26—H26B	109.9
N1—C8—H8A	109.7	H26A—C26—H26B	108.3
C7—C8—H8A	109.7	C28—C27—C32	117.47 (14)
N1—C8—H8B	109.7	C28—C27—C26	122.76 (14)
C7—C8—H8B	109.7	C32—C27—C26	119.77 (14)
H8A—C8—H8B	108.2	F4—C28—C27	118.67 (14)
N1—C9—C10	110.27 (12)	F4—C28—C29	117.60 (15)
N1—C9—H9A	109.6	C27—C28—C29	123.74 (15)
C10—C9—H9A	109.6	C30—C29—C28	117.02 (16)
N1—C9—H9B	109.6	C30—C29—H29	121.5
C10—C9—H9B	109.6	C28—C29—H29	121.5
H9A—C9—H9B	108.1	F5—C30—C29	120.43 (16)
C11—C10—C15	117.22 (14)	F5—C30—C31	118.55 (16)
C11—C10—C9	122.38 (14)	C29—C30—C31	121.00 (15)
C15—C10—C9	120.36 (14)	F6—C31—C32	119.75 (15)
F1—C11—C10	118.92 (15)	F6—C31—C30	119.01 (14)
F1—C11—C12	117.63 (14)	C32—C31—C30	121.24 (15)
C10—C11—C12	123.44 (15)	C31—C32—C27	119.54 (15)
C13—C12—C11	117.47 (15)	C31—C32—H32	120.2
C13—C12—H12	121.3	C27—C32—H32	120.2
C11—C12—H12	121.3	C24—C33—C34	133.41 (15)
F2—C13—C12	119.91 (15)	C24—C33—H33	113.3
F2—C13—C14	118.98 (15)	C34—C33—H33	113.3
C12—C13—C14	121.11 (15)	O8—C34—O7	121.92 (14)
F3—C14—C13	118.81 (14)	O8—C34—C33	127.55 (14)
F3—C14—C15	120.53 (14)	O7—C34—C33	110.48 (14)
			
O2—S1—N1—C8	165.08 (10)	C11—C10—C15—C14	0.3 (2)
O1—S1—N1—C8	−65.38 (11)	C9—C10—C15—C14	178.24 (14)
C1—S1—N1—C8	47.13 (11)	C6—C7—C16—C17	−4.9 (3)
O2—S1—N1—C9	28.63 (13)	C8—C7—C16—C17	169.70 (15)
O1—S1—N1—C9	158.17 (11)	C7—C16—C17—O4	−7.5 (3)
C1—S1—N1—C9	−89.32 (12)	C7—C16—C17—O3	174.59 (16)
O6—S2—N2—C25	−164.94 (10)	O6—S2—C18—C19	−49.22 (15)
O5—S2—N2—C25	65.20 (11)	O5—S2—C18—C19	81.24 (13)
C18—S2—N2—C25	−48.23 (11)	N2—S2—C18—C19	−165.08 (12)
O6—S2—N2—C26	−28.22 (13)	O6—S2—C18—C23	132.05 (13)
O5—S2—N2—C26	−158.09 (11)	O5—S2—C18—C23	−97.48 (13)
C18—S2—N2—C26	88.48 (12)	N2—S2—C18—C23	16.19 (14)
O2—S1—C1—C2	48.83 (14)	C23—C18—C19—C20	0.8 (2)
O1—S1—C1—C2	−80.91 (13)	S2—C18—C19—C20	−177.93 (13)
N1—S1—C1—C2	165.80 (12)	C18—C19—C20—C21	0.6 (3)
O2—S1—C1—C6	−136.40 (12)	C19—C20—C21—C22	−0.6 (2)
O1—S1—C1—C6	93.86 (13)	C20—C21—C22—C23	−1.0 (2)
N1—S1—C1—C6	−19.43 (14)	C21—C22—C23—C18	2.3 (2)
C6—C1—C2—C3	−4.0 (2)	C21—C22—C23—C24	180.00 (14)
S1—C1—C2—C3	170.69 (12)	C19—C18—C23—C22	−2.2 (2)
C1—C2—C3—C4	−2.9 (2)	S2—C18—C23—C22	176.43 (11)
C2—C3—C4—C5	5.6 (2)	C19—C18—C23—C24	−179.98 (14)
C3—C4—C5—C6	−1.4 (2)	S2—C18—C23—C24	−1.3 (2)
C4—C5—C6—C1	−5.3 (2)	C22—C23—C24—C33	18.1 (2)
C4—C5—C6—C7	175.82 (14)	C18—C23—C24—C33	−164.34 (16)
C2—C1—C6—C5	8.0 (2)	C22—C23—C24—C25	−160.80 (13)
S1—C1—C6—C5	−166.50 (11)	C18—C23—C24—C25	16.81 (19)
C2—C1—C6—C7	−173.07 (14)	C26—N2—C25—C24	−67.74 (16)
S1—C1—C6—C7	12.41 (19)	S2—N2—C25—C24	70.24 (14)
C5—C6—C7—C16	−37.3 (2)	C33—C24—C25—N2	128.86 (14)
C1—C6—C7—C16	143.83 (16)	C23—C24—C25—N2	−52.10 (17)
C5—C6—C7—C8	147.93 (14)	C25—N2—C26—C27	−77.84 (15)
C1—C6—C7—C8	−30.93 (19)	S2—N2—C26—C27	147.39 (11)
C9—N1—C8—C7	67.11 (16)	N2—C26—C27—C28	117.32 (16)
S1—N1—C8—C7	−70.46 (14)	N2—C26—C27—C32	−61.75 (19)
C16—C7—C8—N1	−115.16 (15)	C32—C27—C28—F4	179.40 (14)
C6—C7—C8—N1	60.14 (16)	C26—C27—C28—F4	0.3 (2)
C8—N1—C9—C10	76.81 (16)	C32—C27—C28—C29	−0.7 (2)
S1—N1—C9—C10	−148.39 (11)	C26—C27—C28—C29	−179.81 (16)
N1—C9—C10—C11	−124.09 (16)	F4—C28—C29—C30	−179.77 (15)
N1—C9—C10—C15	58.04 (19)	C27—C28—C29—C30	0.3 (3)
C15—C10—C11—F1	178.93 (14)	C28—C29—C30—F5	178.65 (15)
C9—C10—C11—F1	1.0 (2)	C28—C29—C30—C31	0.1 (3)
C15—C10—C11—C12	−0.6 (2)	F5—C30—C31—F6	0.6 (2)
C9—C10—C11—C12	−178.53 (15)	C29—C30—C31—F6	179.15 (15)
F1—C11—C12—C13	−179.90 (15)	F5—C30—C31—C32	−178.77 (14)
C10—C11—C12—C13	−0.4 (3)	C29—C30—C31—C32	−0.2 (3)
C11—C12—C13—F2	−178.60 (14)	F6—C31—C32—C27	−179.54 (13)
C11—C12—C13—C14	1.7 (2)	C30—C31—C32—C27	−0.2 (2)
F2—C13—C14—F3	−2.4 (2)	C28—C27—C32—C31	0.6 (2)
C12—C13—C14—F3	177.31 (15)	C26—C27—C32—C31	179.73 (14)
F2—C13—C14—C15	178.23 (14)	C23—C24—C33—C34	1.9 (3)
C12—C13—C14—C15	−2.1 (2)	C25—C24—C33—C34	−179.25 (15)
F3—C14—C15—C10	−178.34 (14)	C24—C33—C34—O8	14.6 (3)
C13—C14—C15—C10	1.0 (2)	C24—C33—C34—O7	−168.18 (17)

### Hydrogen-bond geometry (Å, º)

**Table:**

*D*—H···*A*	*D*—H	H···*A*	*D*···*A*	*D*—H···*A*
O3—H3*O*···O8^i^	0.85 (3)	1.86 (3)	2.7063 (17)	172 (3)
O7—H7*O*···O4^ii^	0.92 (3)	1.73 (3)	2.6473 (17)	177 (2)
C15—H15···O6	0.95	2.34	3.267 (2)	165
C32—H32···O2	0.95	2.61	3.539 (2)	165

Symmetry codes: (i) *x*+1, *y*, *z*−1; (ii) *x*−1, *y*, *z*+1.
